# An Efficient and Secure Revocation-Enabled Attribute-Based Access Control for eHealth in Smart Society

**DOI:** 10.3390/s22010336

**Published:** 2022-01-03

**Authors:** Shahzad Khan, Waseem Iqbal, Abdul Waheed, Gulzar Mehmood, Shawal Khan, Mahdi Zareei, Rajesh Roshan Biswal

**Affiliations:** 1 Department of Information Security, Military College of Signals (MCS), NUST, Islamabad 44000, Pakistan; skhan.phdismcs@student.nust.edu.pk (S.K.); waseem.iqbal@mcs.edu.pk (W.I.); 2 Department of Computer Science, Shaheed Benazir Bhutto University Sheringal, Dir 18000, Pakistan; 3 Department of Computer Science, Northern University, Nowshera 24100, Pakistan; 4 School of Electrical and Computer Engineering, Seoul National University, Seoul 08826, Korea; 5 Department of Computer Science, IQRA National University, Swat Campus 19220, Pakistan; gulzar.mahmood@uom.edu.pk; 6 Department of Computer Science, COMSATS University, Islamabad 44000, Pakistan; shawalsbbu@gmail.com; 7 Tecnologico de Monterrey, School of Engineering and Sciences, Zapopan 45201, Mexico; m.zareei@tec.mx (M.Z.); rroshanb@tec.mx (R.R.B.)

**Keywords:** access control, attribute-based encryption, elliptic curve, wireless body area networks, smart society

## Abstract

The ever-growing ecosystem of the Internet of Things (IoT) integrating with the ever-evolving wireless communication technology paves the way for adopting new applications in a smart society. The core concept of smart society emphasizes utilizing information and communication technology (ICT) infrastructure to improve every aspect of life. Among the variety of smart services, eHealth is at the forefront of these promises. eHealth is rapidly gaining popularity to overcome the insufficient healthcare services and provide patient-centric treatment for the rising aging population with chronic diseases. Keeping in view the sensitivity of medical data, this interfacing between healthcare and technology has raised many security concerns. Among the many contemporary solutions, attribute-based encryption (ABE) is the dominant technology because of its inherent support for one-to-many transfer and fine-grained access control mechanisms to confidential medical data. ABE uses costly bilinear pairing operations, which are too heavy for eHealth’s tiny wireless body area network (WBAN) devices despite its proper functionality. We present an efficient and secure ABE architecture with outsourcing intense encryption and decryption operations in this work. For practical realization, our scheme uses elliptic curve scalar point multiplication as the underlying technology of ABE instead of costly pairing operations. In addition, it provides support for attribute/users revocation and verifiability of outsourced medical data. Using the selective-set security model, the proposed scheme is secure under the elliptic curve decisional Diffie–Hellman (ECDDH) assumption. The performance assessment and top-ranked value via the help of fuzzy logic’s evaluation based on distance from average solution (EDAS) method show that the proposed scheme is efficient and suitable for access control in eHealth smart societies.

## 1. Introduction

**Figure 1:**
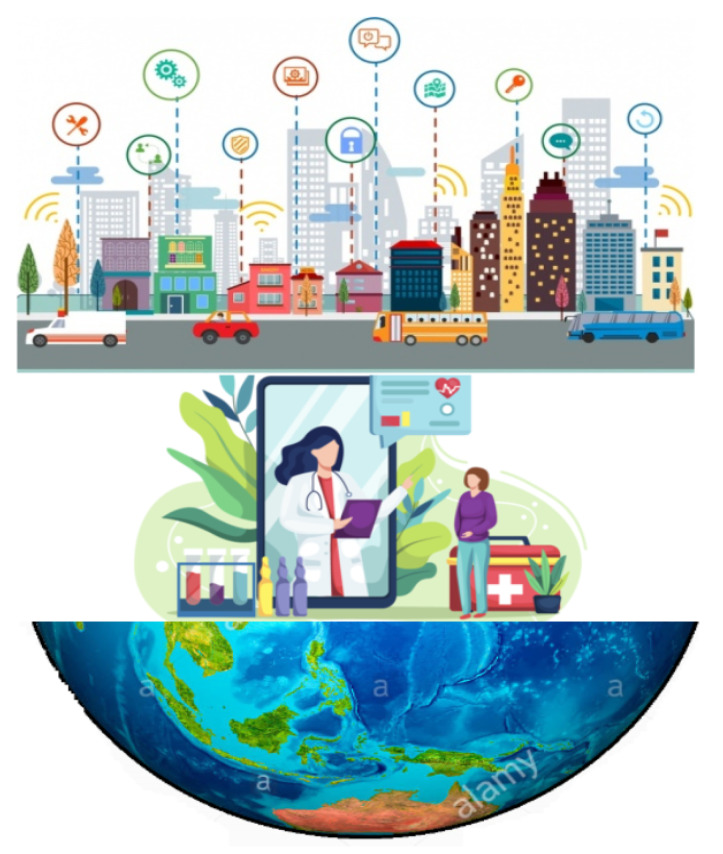
eHealth in smart societies.

The transformative effect of eHealth on smart society (shown in Figure 1) enables wearable medical devices for a vast number of applications, such as wearable fitness trackers, smart health watches, electrocardiogram (ECG) monitors, blood presser monitors, biosensors, etc. On the other front, advances in wireless communication lead to the emergence of the solidified and specialized wireless area network for these worn-on or implanted devices; the wireless body area network (WBAN). A WBAN typically consists of tiny biosensors or sensors (wearable and/or implanted) to collect/forward vital signs to the mobile or fixed gateway. It was developed to enable around-the-clock availability of a patient’s medical data to healthcare professionals. This unremitting availability of data will efficiently utilize healthcare resources and makes in-home monitoring for patients having chronic diseases [1]. Unlike conventional sensor networks, a WBAN operates on more critical and sensitive patient information that demands significant security and privacy preservation from the practical aspect of this technology. This concern leads to the desire for more control of their data from the data owner end. This self-contradicting aspect results in severe security challenges for its practical adaptation. In the presence of its underlying Internet of Things (IoT) infrastructure, conventional encryption techniques preclude its adaption for WBAN security. Specifically, public-key encryption suffers from high computation, certificate, and key management overhead issues. The dynamic secret key management hinders the application of symmetric encryption as well. Considering the nature of WBAN healthcare systems, it is inevitable to provide this crucial data to its concerned healthcare professionals. Hence, traditional role-based access control and identity-based encryption (IBE) cannot guarantee fine-grained and one-to-many data transfer. Recently, attribute-based encryption (ABE) has gained popularity for secure access control mechanisms to confidential data because of its inherent support for fine-grained access and one-to-many transfer. ABE is a particular type of IBE; the user’s ID is described by the set of attributes, in which the data is encrypted for all those users who are the possessors of that specific set of attributes. The ABE schemes are categorized into two variants: ciphertext policy (CP-ABE) and key-policy (KP-ABE). Using CP-ABE, the data owner embeds access policy inside ciphertext and the private key of the end user is attached to the attribute set. Anyone can perform the decryption operation if his/her attributes matched with the specified access policy. While in KP-ABE, private keys are attached with the access control policy and ciphertext are attached with the attribute set [2]. In the context of WBAN, ciphertext policy ABE (CP-ABE) is more appropriate because it provides more control to the data owners (patient in WBAN) over the recipients [3] (medical stuff in WBAN) as opposed to its other type, i.e., key-policy ABE (KP-ABE) [4]. The only series concern for most contemporary ABE schemes is that they rely heavily on expensive bilinear pairing and exponentiation operation in the encryption and decryption algorithm. This intense computation hinders its deployment for WBAN resource-constrained sensors [3,5]. This leads to the development of non-pairing ABE schemes in the research community. As a result, the most recent work equips the ABE with the elliptic curve cryptography (ECC) algorithms, which have much stronger bit security and also replace the ten times more expensive bilinear pairing operation with scalar point multiplication on an elliptic curve [3]. At the same time, because of underlying ABE technology, linearity properties entrust the ECC algorithm with heavy operations. As we know, the number of operations linearly increases with the number of attributes and hence incurs a heavy load on WBAN sensors. Therefore, a secure and efficient management mechanism is needed, which stands this operation to an acceptable and minimum constant range for WBAN sensors nodes. In this paper, by utilizing Hu et al.’s [4] secure framework for WBAN, we have proposed an efficient and secure ECC-based CP-ABE scheme for WBAN.

### Our Contribution

The primary contribution of our work is as follows:

1. Considering the resource-scarce nature of WBAN, we have proposed an efficient and secure ABE scheme with outsourcing intense encryption and decryption operations without revealing the secret key/data content to the WBAN data sink node and cloud server digital signal processing (DSP), respectively.
2. Our proposed scheme is based on elliptic curve point scalar multiplication instead of costly bilinear pairing operations to address the resource-constrained nature of WBAN, especially the sensors. This feature makes it more appealing to smart healthcare.
3. Our proposed scheme supports indirect attribute/users revocation without the need for maintaining a private channel between the trusted attribute authority and the non-revoked users for disseminating updated decryption keys.
4. The proposed scheme inherently supports the integrity check, thus increasing the security and reliability of medical data.
5. The proposed scheme is secure under the elliptic curve decisional Diffie–Hellman (ECDDH) assumption using the selective-set security model.
6. The performance assessment of our scheme shows a significant overall efficiency in storage, computation, and communication.

## 2. Related Work and Background Knowledge

This section presents a brief overview of existing work and all the cryptographic primitives used to construct our proposed scheme.

### 2.1. Related Work

With the emerging use of e-healthcare systems, patients are not only concerned for the security of their personal information but also worry for the privacy of their biological characteristics [6,7]. To improve the performance, early approaches utilized cloud computing models for e-healthcare systems. For example, in [8] they have proposed a patient-oriented four-layer cloud-based e-healthcare system. With the emergence of edge computing and its proximity to resource-constrained devices, many edge-based e-healthcare systems [9,10] are proposed. In [11], the author developed a first-aid service to provide emergency aid to the patients rapidly. However, the early approach lacks the much-needed security requirements. For the realization of security in smart healthcare, the author in [12] utilizes fully homomorphic encryption (FHE) to encrypt the data. For better security, Cai et al. [13] create a novel medical record based on the mobile cloud without compromising too much performance. Still, the above system does not devise any proper access control mechanism for these medical records. So, to better protect data privacy, some schemes equipped with access control were proposed [14,15]. For example, in [15], the author suggests a role-based access control with the capability of origin tracing and further scrutinizing the authorization of access made to the system resources. However, fine-grained access control is needed for better and flexible access, which requires the exposure of specific portions of data to the relevant medical professionals. Attributed to its inherent expressiveness and fine-grained access support, attribute-based encryption (ABE) has emerged. Sahi and waters [16] were the first to interpret the identity of users as a set of attributes and were able to propose a fuzzy variation of identity-based encryption (IBE). Attributed to the placement of access policy, ABE has two variations, namely key-policy ABE (KP-ABE) [17] and ciphertext policy ABE (CP-ABE) [18]. Li et al. [19] propose the outsourcing of encryption with MapReduce to relieve local computation overhead. Li et al. [20] construct a novel ABE scheme which outsourced both the key-issuing and decryption with the verification of the results returned from the cloud server. Asim et al. [21], with the help of a semi-trusted proxy, outsourced the computation of message encryption by utilizing the El-Gamal cipher. However, the scheme is proven in the generic group model. Zong et al. [22] utilize the edge-enabled environment for outsourcing part of encryption and decryption to the edge node for the smart healthcare system. Zhidan et al. [23] propose the construction of an ABE scheme with verifiable delegation both for encryption and decryption to an untrusted encryption service provider (ESP) and a decryption service provider (DSP), respectively. Khan et al. [24] propose an online/offline-aided attribute-based multi-keyword search (OOABMS) scheme to delegate most heavy computation operations to the offline phase before acquiring the attribute-based access control policy or keywords. However, all of these ABE schemes were heavily dependent on a costly bilinear pairing operation [25]. Later, in [26], the author proposed a free-pairing lightweight KP-ABE scheme using ECC for resource constraint of IoT infrastructure. Consequently, Tan et al. [27] introduces the concept of key out-sourcing property in [26] for better efficiency without compromising its security. Several body sensor network (BSN) [28,29], are proposed for the cloud environment that exhibits their usability and favorability for the key-policy type of ABE in different scenarios. KP-ABE transferred the computation overhead of access policy formulation to the medical attribute authority (MAA) from the patient but at the same time offered no control over it. CP-ABE offers complete control over who has access to the sensitive medical data, making it conceptually similar to the role-based access control [30] model.

These appealing characteristics for WBAN resulted in the basis for many proposed [18] schemes with various features such as policy update, hidden access policy, traceability, and revocability. These schemes mainly utilized costly pairing operations. Considering the resource constraint nature of a WBAN, pairing-free ABE schemes should be the first choice of a WBAN. In this direction, Ref. [31] proposed a pairing-free ECC-based CP-ABE scheme. However, similar to most of the schemes, this also suffers from the inherent linearity property of ABE. For the sake of practical deployment, we have designed a pairing-free CP-ABE scheme based on ECC with a minimal constant number of scalar point multiplication.

Basar et al. [32] present an image segmentation method based on pulse coupled neural network (PCNN) and local binary pattern (LBP) components. The proposed method is robust because the presented model’s parameters can be modified for different situations. The proposed algorithm has been tested on a dataset that consists of 1000 defocused images. The results show that the proposed algorithm outperforms contemporary algorithms on different evaluation metrics such as accuracy and precision. A fuzzy logic-based ranking based on EDAS has been used for ranking. The experimental results and evaluation show that the proposed scheme outperforms contemporary schemes in terms of time complexity and accuracy.

Mehmood et al. [33] developed a trust-based energy-efficient and reliable communication scheme named trust-based ERCS for remote patient monitoring in eHealth applications. A cooperative communication strategy is used in the proposed scheme to ensure trust and reliability. Furthermore, privacy preservation and a fuzzy-logic rank-based method have been used in the proposed scheme. The detailed experimental results and ranking demonstrated that the proposed scheme outperforms the available contemporary schemes.

Similarly, Basar et al. [34] present a method for an RGB histogram-based K-means clustering initialization for unsupervised color image segmentation. In this method, an adaptive initialization approach has been used to determine the number of clusters and initial central points of each cluster to solve the segmentation issues of color images. The proposed method is compared with well-known unsupervised segmentation methods on various segmentation parameters. Furthermore, the EDAS (evaluation based on distance from average solution) technique is used to rank segmentation integrity. The experimental results show that the proposed method outperformed the contemporary methods. However, due to classification errors, the proposed method is not recommended for healthcare medical applications.

### 2.2. Background Knowledge

This section presents all the cryptographic primitives used for the construction of our proposed ECC-based ABE scheme, including elliptic curve cryptosystem, lagrange interpolation for secret reconstruction, and access control structure.

### 2.3. Elliptic Curve Cryptosystem and Its Related Complexity Assumptions

An elliptic curve *E* over a prime finite field $Z_{p}$ is defined by a cubic equation

$$y^{2}(mod\;p)=x^{3}+a\cdot x+b(mod\;p)$$

while the set of parameters (p,a,b,G,n) can be used for its description, where $x,y,a,b\in Z_{p}$, and $4a^{3}+27b^{2}\neq 0$. All the point operations in ECC must be define to form a cyclic group $G_{E}$ over E.

**Definition** **1** (Elliptic curve discrete logarithm problem (ECDLP)). *Given points P and Q on the curve, i.e., $P,Q,\in G_{E}$, it is intractable for a polynomial time algorithm to get the random chosen value $K\in Z_{q}^{*}$ such that Q=K∗P.*

**Definition** **2** (Elliptic curve computational Diffie–Hellman problem (ECCLP)). *For generator G of $G_{E}$ and randomly chosen values $c,d,\in Z_{q}^{*}$, given (c·G,d·G,G) it is intractable for a polynomial time algorithm to get c·d·G.*

**Definition** **3** (Elliptic curve decisional Diffie–Hellman problem (ECDLP)). *Given randomly chosen values $c,d\in Z_{q}^{*}$ and generator G and any point Z of $G_{E}$, it is impossible to distinguish between the two probability distributions (c·G,d·G,c·d·G) and (c·G,d·G,Z).*

**Definition** **4** (Access tree). *Access tree [17]. Let a tree T represent an access structure. Each non-leaf node of T is identified by a threshold gate, associated by its corresponding threshold value and its children. In this case, if $d_{x}$ is the threshold value of node x and $num_{x}$ is its number of children, then $1\leq d_{x}\leq num_{x}$. When $d_{x}=num_{x}$, the threshold gate is an AND gate, and when $d_{x}=1$, it is an OR gate. Each leaf node x of T is identified by a threshold $d_{x}=1$ value and an attribute. Further, definitions and notations can be obtained from [35].*

*In ABE, the lagrange interpolation is used for secret reconstruction. The lagrange coefficient ${▵}_{i,s}$ for a random number in $Z_{p}^{*}$ and a set of random elements corresponding to each element in $Z_{p}^{*}$ is given by ${▵}_{i,s(x)}=\Pi_{j\in s,j\neq i}\frac{x-j}{i-j}$.*

## 3. System and Security Model

**Figure 2:**
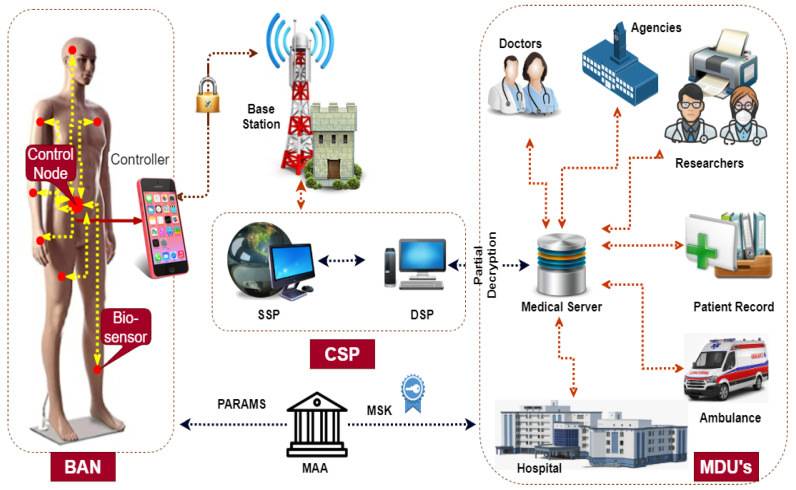
System model.

Figure 2 depicts the main components of our proposed scheme, namely the medical attribute authority (MAA), cloud service provider (CSP), body area network (BAN), data sink (DS), and medical data user (MDU). This section presents an overview of the roles played by each component.

**MAA:** The MAA acts as a key generation center (KGC) and the only fully trusted entity in the system model. KGC is responsible for the registration of all system users [36]. Through the initialization phase, it produces public parameters (PARAMS), a system master key (SMK), and secret key components (SK) against a set of attributes $S_{u}$ specific to each user.

**CSP:** This entity is providing services for storage and partial decryption via sub-entities storage service provider (SSP) and decryption service provider (DSP), respectively. The SSP stores the encrypted health-related data for each registered patient and serves as a repository for all the uploaded data. DSP performs partial decryption service to the interested MDU’s without knowing the actual data contents.

**BAN:** Body area network is a wireless network consisting of small biosensors. It could be implanted (placed inside the human body), wearable (on the body), or carried based on its specific use. Its deployment aims to persistently measure and notice the abnormal changes in the vital body parameters. Subsequently, consult in real time the healthcare professional for life support. Sensors are suffering from a scarcity of vital resources in memory, battery power, and computation power. In the traditional framework, these [31] resource-constrained sensors are entrusted with the expensive secret distribution mechanism for access formulation along with its prime tasks of sensing, processing, and transmission. Moreover, because of the ABE linearity property, the encryption complexity grows with the size of the access policy. Exploiting the delegation property of the CP-ABE mode of encryption, we offload most of the computation to the gateway. More specifically, retaining part of the secret for little processing locally while exposing part of it to the gateway for most processing still ensures information-theoretical security of a secret.

**DS:** DS acts as a gateway for aggregation and dissemination of its corresponding sensor data to the MAA. It could be a mobile device such as a smartphone or a specialized BAN controller. Hence, it has significantly more memory, processing, and transmission capacity as opposed to the sensors. These features make us compel in our proposed framework to delegate most of the processing overhead from sensors to the DS. The traditional framework [31] devotes this unit to the function of forwarding only, which is not a judicious use of this entity considering its resources.

**MDU:** It could be a doctor, nurse, or any other healthcare expert. To be registered into the system, each MDU must prove its credentials and affiliation in a set of attributes to the KGC. The KGC needs to verify the validity of these claimed attributes, subsequently computes its corresponding secret key components, and sends it via a secure channel to its concerned user. These secret key components are uniquely generated to prevent collision attacks by associating a random number to them. As long as the MDU poses the required set of attributes, it can access any patient’s encrypted data. MDU is usually a device, such as a mobile phone, with limited resources. In our framework, we shift most of the decryption overhead to the DSP of MAA. As a result, after retrieving the partially encrypted data from the DSP, it needs to perform a minor operation on its full decryption.

In our threat model, we take the CSP honest-but-curious, adapted by most of the ABKS schemes, which means they will honestly run the algorithm and infer privacy information based on the available data. The medical attribute authority and the data owner (DO) are fully trusted entities in our system model. Corrupted data users (DU) may also collide with each other. To prove the security of an ABE scheme, the selective-set security model generally makes use of a game between the challenger C and an attacker A. In this game, the attacker faces challenges posed by the challenger to solve the underlying security assumption. Following are the six steps defined in our security game for our proposed scheme against a chosen-plaintext attack [35].

**Initialization:**A declares the encryption attribute set in the form of an access structure $T^{*}$ that he wants to be challenged upon.

**Setup:** To generates the system parameters, C runs the setup algorithm, keeps the SMK to itself and sends the public parameter PARAMS to adversary A.

**Phase 1:** The adversary A is allowed to adoptively ask for a set of secret key components $K_{A}^{1},K_{A}^{2},\ldots ,K_{A}^{n}$ of attribute sets ${℧}_{1},{℧}_{2},\ldots ,{℧}_{n}$ such that all the attribute sets associated to the corresponding secret key components do not satisfy the $T^{*}$.

**Challenge:** Now, A submits two equal length messages $M_{0}$ and $M_{1}$ to C with $T^{*}$. C flips binary coin *b* ∈{0,1} to encrypt $M_{b}$ under $T^{*}$ and sends the generated ciphertext $CT^{*}$ to A.

**Phase 2:** Both adversary A and challenger C adoptively repeat the same steps as they did in phase 1.

**Guess:**A outputs a guess $b^{\prime }$ of b to C.

The advantage ϵ gained by A in the above game is defined by $\epsilon =(pr\left[b^{\prime }=b\right]-\frac{1}{2})$.

Table 1 lists all the notations used in this work.

## 4. Proposed Model

In this section, a detail description of our proposed scheme algorithms (i.e., Setup, KeyGeneration, $Encrypt_{local}$, $Encrypt_{esp}$, $Decrypt_{dsp}$, $Decrypt_{local}$) is presented.

**Setup (λ)→PK,MK:** Run by MAA, the Algorithm 1 takes EEC domain parameters as an implicit security parameter λ as input. Define the universal attribute set $U=\{att_{1},att_{2},\ldots att_{n}\}$ for attribute space in the system. A secure hash function $H:{\left\{0,1\right\}}^{*}\to Z_{q}^{*}$ is chosen to map global identity GID. MAA for each attribute $att_{i}\in U$, chooses $\beta_{i}\in Z_{p}^{*}$ uniformly at random. The public key components corresponding to each system attribute $att_{i}$ is given by $PK_{i}=\beta_{i}\cdot G$. Moreover, it chooses $\alpha \in Z_{p}^{*}$ uniformly at random to be the master secret key (MSK). Thereafter, setting accordingly, the master public key (MPK) is PK=α·G. Finally, the algorithm sets the $MSK=(\alpha ,\beta_{i}|i\in U)$ and $PARAMS=(U,H,PK,\left\{PK_{i}|i\in U\right\})$.
**Algorithm 1:** Setup (λ).**Input** Implicit security parameter λ.**Output** System secret key (SMK) and public parameter.Define an elliptic curve E over a finite field $Z_{r}$ with a prime order *r*.Generate a cyclic group $G_{E}$ of subgroup over E with generator *G* of order *q*.Generate universal attribute set $U=\{att_{1},att_{2},\ldots att_{n}\}$.For each $att_{i}\in U$, it randomly chooses element $\beta_{i}\in Z_{q}^{*}$.MAA subsequently computes public key components corresponding to each attribute *i* as $\left\{PK_{i}=\beta_{i}\cdot G|i\in U\right\}$.Randomly chooses $\alpha \in Z_{q}^{*}$ as a master secret key.Accordingly, compute master public key by PK=α·G.Set the $PARAMS=(U,H,PK,\left\{PK_{i}=\beta_{i}\cdot G|i\in U\right\})$.Set the $MSK=(\alpha ,\left\{\beta_{i}|i\in U\right\})$.

   **Encryption:** To preserve the data privacy and delegate most of the computation of encryption, this algorithm specifies the access control policy tree in the form of $T=T_{local}⋀T_{esp}$, where $T_{local}$ and $T_{esp}$ are two subtrees of T connected by an AND logical operator ⋀. This division of access control tree leads to two algorithms: local encryption (Algorithm 2) and outsource encryption (Algorithm 3).

**Figure 3:**
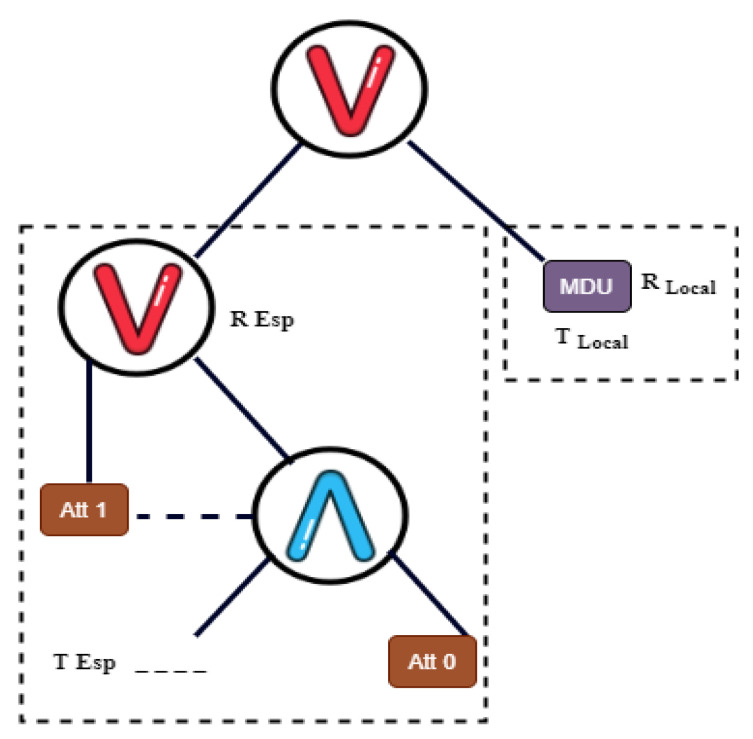
Access policy with subtree.

${\mathrm{Encrypt}}_{\mathrm{local}}$$\left(T,M,PK\right)\to CT_{local}$ For optimal efficiency, the $T_{local}$ attaches only one virtual attribute, as shown in Figure 3. The algorithm randomly specify a 1-degree polynomial $q_{R}\left(\cdot \right)$ and set $q_{R}\left(0\right)=S$, $q_{R}\left(1\right)=S_{1}$ and $q_{R}=S_{2}$, where $S,S_{1},S_{2}\in Z_{q}^{*}$.

Let $\Omega_{local}$ be the set of leaf nodes in $T_{local}$. This algorithm encrypts *M* by computing $SK=S\cdot PK=(S_{x},S_{y})$ such that SK≠0. Let $S_{x}$ serve as the encryption key and $S_{y}$ be the integrity key for *M*, then $C_{M}$ and $INT_{M}$ can be computed $Enc(S_{x},M)$ and $HMAC(S_{y},M)$, respectively. Finally, the algorithm outputs temporal ciphertext

$$CT_{local}=\left(T_{local},C_{M},INT_{M},\forall y\in \Omega_{local}:C_{y}=q_{y}\left(0\right)\cdot PK_{y}\right).$$

$${\mathrm{Encrypt}}_{\mathrm{ESP}}(T_{esp},s_{1},CT_{local},PK)\to CT.$$

Let $\Omega_{ESP}$ be the set of leaf nodes in $T_{esp}$. Beginning at the root node $R_{1}$ of the subtree $T_{esp}$, this algorithm chooses a polynomial $q_{x}$ of degree $d_{x}-1$ for each node *v*. Note that the value for root node $R_{1}$ has been set as $q_{R_{1}}\left(1\right)=S_{1}$. The value of the inner node *x* is calculated by the equation as $q_{x}\left(0\right)=q_{parent(x)}\left(index\left(q\right)\right)$ and randomly chooses $k_{x}-1$ coefficients to build the polynomial $q_{x}$. Then, the algorithm generates the temporal ciphertext $CT_{ESP}=\left(T_{esp},\forall y\in \Omega_{ESP}:C_{y}=q_{x}\left(0\right)\cdot PK_{y}\right)$. Combining the above generated ciphertext with the received ciphertext from DO, the whole ciphertext is given as:

$$\langle T=T_{local}⋀T_{esp};C_{M};INT_{M};\forall y\in \Omega_{local}⋃\Omega_{ESP}:C_{y}=q_{y}\left(0\right)\cdot PK_{y}\rangle$$

**Key Generation**$\left(S_{u},MSK\right)\to K_{u}$ The Algorithm 4 runs by MAA, and is used to generate the secret key $K_{u}$ under the valid attribute set $S_{u}$ by the corresponding DU. More specifically, upon receiving the claimed attribute set, the MAA needs to check its validity and assign a unique global identity GID to this DU. It selects a random $t\in Z_{p}^{*}$ and computes local private key $K_{local}=\alpha^{1-t}$. This algorithm for each attribute $i\in S_{u}$ generates its corresponding key components, a delegate key given by $\mathrm{DK}=\{\forall i\in S_{u}:K_{i}=H\left(GID\right)\cdot \alpha .\beta_{i}^{-1}\}$. Here, $\beta_{i}^{-1}$ is the inverse of element $\beta_{i}\in Z_{p}^{*}$ chosen in setup phase.
**Algorithm 2:**$Encrypt_{local}$.**Input** Access structure T, the message *M* and public parameters PARAMS.**Output** Local version of ciphertext $CT_{local}$.Randomly specify a 1-degree polynomial $q_{R}\left(x\right)$ corresponding to the root *R* of T.Randomly chooses $S,S_{1}$ and $S_{2}\in Z_{q}^{*}$.Set the root node *R* value to $q_{R}\left(0\right)=S$.For the root nodes $R_{1}$ and $R_{2}$ of subtrees set $q_{R}\left(1\right)=S_{1}$ and $q_{R}\left(2\right)=S_{2}$,respectively.Use ECC scalar point multiplication to compute $S\cdot PK=(S_{x},S_{y})$. We let $S_{x}$and $S_{y}$ represent the encryption and integrity key for *M*, respectively.Compute message *M* encryption $C_{M}=Enc\left(S_{x},M\right)$ using secure symmetriccipher.Compute message *M* authentication code $INT_{M}=HMAC\left(S_{y},M\right)$ using HMAC function.Let $\Omega_{local}$ be a set of leaf nodes in $T_{local}$.**For** each $att_{x}\in \Omega_{local}$ do.$CT_{local}=q_{x}\left(0\right).PK_{x}$ using ECC point multiplication **End for**.Set the ciphertext $CT_{local}=\left(T_{local},C_{M},INT_{M},\forall y\in \Omega_{local}:C_{y}=q_{y}\left(0\right)\cdot PK_{y}\right)$.

**Algorithm 3:**$Encrypt_{ESP}$.
**Input** Access structure $T_{esp},S_{1},CT_{local}$, and public parameters PARAMS.
**output**CT.
Randomly specify a polynomial $q_{R_{1}}$ with degree $K_{R_{1}}-1$, where $K_{R_{1}}$ is the thresholdof root node of subtree $T_{ESP}$.Set the value of root node $R_{1}$ to $q_{R_{1}}\left(0\right)=S_{1}$.Randomly select $K_{R_{1}}-1$ coefficients to uniquely define $q_{R_{1}}$.**For** inner node *v* in $T_{esp}$ do.Set $q_{v}\left(0\right)=q_{parent(v)}\left(index\left(v\right)\right)$.Randomly select $K_{v}-1$ coefficients to uniquely define $q_{v}$.**End For**.Let $\Omega_{ESP}$ be the set of leaf nodes in $T_{esp}$.**For** each $att_{x}\in \Omega_{ESP}$ do.$CT_{ESP}=q_{x}\left(0\right).PK_{x}$ using ECC point multiplication.**End For**.The whole ciphertext is given by $CT=\langle T=T_{local}⋀T_{esp},C_{M},INT_{M},\forall y\in \Omega_{local}⋃\Omega_{ESP}:C_{y}=q_{y}\left(0\right)\cdot PK_{y}\rangle$.


**Algorithm 4:**KeyGen.
Input DU claimed attribute set $S_{u}$, system master key SMK Output DU keys: $K_{local}$ and DK.
After the confirmation of the claimed attribute set $S_{u}$, the MAA assigned a global unique identity GID to its DU.Select a random $t\in Z_{p}^{*}$, compute $\alpha^{t}$.Compute and set $K_{local}=\alpha^{1-t}$.**For** each $att_{i}\in S_{u}$ do.Compute $\beta_{i}^{-1}$ of $\beta_{i}\in Z_{p}^{*}$.Compute $K_{i}=H\left(GID\right)\cdot \alpha^{t}\cdot \beta_{i}^{-1}$.**End For**.Set the Keys $K_{local}=\left(\alpha^{1-t}\right)$, $\mathrm{DK}=(\left\{\forall i\in S_{u}:K_{i}=H\left(GID\right)\cdot \alpha^{t}\cdot \beta_{i}^{-1}\right\};H\left(GID\right))$.


Finally, the algorithm via a secure channel submits the secret keys $K_{local}=\left(\alpha^{1-t}\right)$ and $\mathrm{DK}=(\left\{\forall i\in S_{u}:K_{i}=H\left(GID\right)\cdot \alpha^{t}\cdot \beta_{i}^{-1}\right\};H\left(GID\right))$ to its concerned DU.

**Decryption:** Realizing a CP-ABE scheme via ECC scalar point multiplication instead of bilinear pairing operations still faces a deployment challenge for lightweight devices, especially for sensors. The ECC scheme makes use of threshold secret sharing for secret distribution. Subsequently, the reconstruction makes use of polynomial interpolation, a heavy computation operation. MDU is usually a device such as a mobile phone with limited resources. Hence, this phase delegates most of the decryption load to the DSP. This phase makes use of two algorithms $Decrypt_{local}$ (Algorithm 5) and $Decrypt_{DSP}$ (Algorithm 6).

${\mathrm{Decrypt}}_{\mathrm{DSP}}\;\left(\mathrm{DK},PARAM,CT\right)\to CT_{temp}$

This algorithm is run by DSP, which makes use of a recursive function DecNod(CT,DK,y). If *y* is leaf node, let i=att(y), DecNode(CT,DK,y) is defined as:

$$DecNode\left(CT,\mathrm{DK},y\right)=\left\{\begin{array}{c} \frac{K_{i}\cdot C_{i}}{H(GID)},\forall i\in S_{u}\\ ⊥,otherwise. \end{array}\right..$$

which states that the output of DecNode() must be an element in EC group $G_{E}$ or null.

For a leaf node $y\in S_{u}$, the function DecNode() proceeds as follows:

$$\begin{array}{c} DecNode\left(CT,\mathrm{DK},y\right)=\frac{K_{i}\cdot C_{i}}{H(GID)}\\ =\frac{H\left(GID\right)\cdot \alpha^{t}\cdot \beta_{i}^{-1}\cdot q_{y}\left(0\right)\cdot PK_{i}}{H(GID)}\\ =\alpha^{t}\cdot \beta_{i}^{-1}\cdot q_{y}\left(0\right)\cdot \beta_{i}\cdot G\\ =q_{y}\left(0\right)\cdot \alpha^{t}\cdot G. \end{array}$$

For a non-leaf node *y*, it calls DecNode() for each child *x* and stores the result as $F_{x}$ in $k_{y}-$ sized set $S_{y}$ of child node *x*. To reconstruct the value of $F_{y}$ at nodes *y* using lagrange interpolation, the algorithm proceeds as follows:

$$F_{y}=\sum_{x\in S_{y}}{▵}_{i,s_{y}^{\prime }}\left(0\right)\cdot DecNode\left(CT,\mathrm{DK},x\right)$$

where $i=index\left(x\right),s_{u}^{\prime }=\left\{index\left(x\right),x\in s_{u}\right\}$ and ${▵}_{i,s_{y}}\left(0\right)$ is the lagrange coefficients

$$\begin{array}{c} =\sum_{x\in s_{y}}{▵}_{i,s_{y}^{\prime }}\left(0\right)\cdot q_{x}\left(0\right)\cdot \alpha^{t}\cdot G\\ =\sum_{x\in s_{y}}{▵}_{i,s_{y}^{\prime }}\left(0\right)\cdot q_{parent(x)}\left(index\left(x\right)\right)\cdot \alpha^{t}\cdot G\\ =\sum_{x\in s_{y}}{▵}_{i,s^{\prime }}\left(0\right)\cdot q_{y}\left(i\right)\cdot \alpha^{t}\cdot G\\ =q_{y}\left(0\right)\cdot \alpha^{t}\cdot G. \end{array}$$

Accordingly, the recursive function DecNode(CT,DK,R) at root node *R* returns $q_{R}\left(0\right)\cdot \alpha^{t}\cdot G$. Finally, the temporal ciphertext $CT_{temp}$ set as: $CT_{temp}=\left\{F_{R}\right\}$.

${\mathrm{Decrypt}}_{\mathrm{local}}\;\left(K_{local},CT_{temp}\right)\to M$. After receiving the intermediate ciphertext $CT_{temp},MDU$ calculates $\left\{F_{R}\right\}\times \mathrm{DK}=q_{R}\left(0\right)\cdot \alpha^{t}\cdot G\times \alpha^{1-t}=q_{R}\left(0\right)\cdot \alpha \cdot G=s\cdot \alpha \cdot G=s\cdot PK=\left(\bar{S_{x}},\bar{S_{y}}\right)$. Here, $\bar{S_{x}}$ and $\bar{S_{y}}$ are the recovered keys for decryption and integrity of message *M*, respectively. Therefore, after decrypting $M^{\prime }=Dec\left(\bar{S_{x}},C_{M}\right)$ we can confirm, whether $HMAC\left(\bar{S_{y}},M^{\prime }\right)=INT_{M}$ to assure that the *M* is correctly received and not being tempered. Hence, the proposed scheme provides confidentiality, authenticity, and integrity of encrypted data, which is the top most priority of any health-related application.
**Algorithm 5:**$Decrypt_{DSP}$.**Input** Delegate key component DK, system public parameter PARAM and CT.  **Out Put** Temporal ciphertext $CT_{temp}$.Let *y* be a node in T.**If**i=att(y) is leaf node AND $i\in S_{u}$ then.Compute $F_{y}=\frac{K_{i}\cdot C_{i}}{H(GID)}$              $=\frac{H\left(GID\right)\cdot \alpha^{t}\cdot \beta_{i}^{-1}\cdot q_{y}\left(0\right)\cdot PK_{i}}{H(GID)}$              $=\alpha^{t}\cdot \beta_{i}^{-1}\cdot q_{y}\left(0\right)\cdot \beta_{i}\cdot G$              $=q_{y}\left(0\right)\cdot \alpha^{t}\cdot G$.**Else**Set $F_{y}=Null$.**End if**.**For** each non-leaf node *y* in T do.Let $s_{y}$ represent $k_{y}$-sized set of child node *x*.**If** no such set exist thenSet $F_{y}=Null$.**Else**Compute lagrange coefficient$F_{y}=\sum_{x\in S_{y}}{▵}_{i,s_{y}^{\prime }}\left(0\right)\cdot DecNode\left(CT,\mathrm{DK},x\right)$       where $i=index\left(x\right),s_{u}^{\prime }=\left\{index\left(x\right),x\in s_{u}\right\}$ and ${▵}_{i,s_{y}}\left(0\right)$ is the lagrange coefficients    $=\sum_{x\in s_{y}}{▵}_{i,s_{y}^{\prime }}\left(0\right)\cdot q_{x}\left(0\right)\cdot \alpha^{t}\cdot G$    $=\sum_{x\in s_{y}}{▵}_{i,s_{y}^{\prime }}\left(0\right)\cdot q_{parent(x)}\left(index\left(x\right)\right)\cdot \alpha^{t}\cdot G$    $=\sum_{x\in s_{y}}{▵}_{i,s^{\prime }}\left(0\right)\cdot q_{y}\left(i\right)\cdot \alpha^{t}\cdot G$    $=q_{y}\left(0\right)\cdot \alpha^{t}\cdot G$.**End if**.**End for**.Let *R* represent the root node of T.**If**$F_{R}\neq Null$ then recursively compute $F_{R}=q_{R}\left(0\right)\cdot \alpha^{t}\cdot G$.**End if** Set the temporal ciphertext $CT_{temp}=\left\{F_{R}\right\}$.

**Algorithm 6:**$Decrypt_{local}$.
**Input**DU local secret key $K_{local}$, and temporal ciphertext $CT_{temp}$.

**Output Message *M*.**

Compute $F_{R}\cdot K_{local}$  $=q_{R}\left(0\right)\cdot \alpha^{t}\cdot G\times \alpha^{1-t}$                     $=q_{R}\left(0\right)\cdot \alpha \cdot G$                     =s·α·G                     =s·PK                     $=(\bar{S_{x}},\bar{S_{y}})$ Decrypt $M^{\prime }=Dec\left(\bar{S_{x}},C_{M}\right)$ and compute $INT_{M}^{\prime }=HMAC\left(\bar{S_{y}},M^{\prime }\right)$.    **If** $INT_{M}^{\prime }=INT_{M}$ then       *M* is valid.     **End if**.     **Return** *M*.


## 5. Security Analysis

This section, along with security proof, also assesses the proposed scheme’s collision resistance and attribute/user revocation features.

### 5.1. Security Proof

The security proof of our scheme in the selective security model is presented as a game between the challenger C and an attacker A. In this game, the attacker confronts challenges posed by the challenger to break the underlying hardness assumption. Since our scheme is based on ECC, hence, the attacker’s goal is to reduce the hardness of the elliptic curve decisional Diffie–Hellman (DDH) assumption.

**Theorem** **1.** *If an adversary A in the selective-set model successfully attacks our proposed scheme with, at most, advantage ϵ, then it can also build a simulator $S_{\beta }$ that can distinguish an elliptic curve DDH tuple with non-negligible advantage $\epsilon^{\prime }$.*

**Proof.**  Let there exist an adversary A, in the particular set security model that in polynomial time with non-negligible advantage ϵ can break our scheme, then we can build a simulator $S_{\beta }$ to play the ECDDH with advantage $\epsilon^{\prime }$ in polynomial time.

Firstly, the challenger C generates an EC group $G_{E}$ with order q and sets over the finite field $Z_{q}^{*}$ having a base point G. Then, challenger C takes a fair binary coin μ∈{0,1}, flips it outside of $S_{\beta }$’s view for some random choices a, b, z $\in Z_{q}^{*}$. Now, the choices for μ is given as:

- Case 1. if μ=0, then ECDDH challenge instance as,
  (A,B,Z)=(c·G,d·G,c·dG), and sent to $S_{\beta }$.
- Case 2. if μ=1, then ECDDH challenge instance as,
  (A,B,Z)=(c·G,d·G,z·G), and sent to $S_{\beta }$.

**Initialization:** The simulator $S_{\beta }$ runs adversary A, to gets an access structure $T^{*}$ that the adversary A wants to be challenged upon.

**Setup:** The simulator $S_{\beta }$ needs to send the public parameters to adversary A as follows:

1. $S_{\beta }$ at first sets the system parameters Y=A=c·G.
2. Then, for ∀∈U, $S_{\beta }$ sets $Y_{i}$ according to the following condition:
  - If i∈℧ it sets $Y_{i}=r_{i}\cdot G$ and $y_{i}=r_{i}$ where $r_{i}$ is randomly chooses from $Z_{q}^{*}$.
  - If i∈(U−℧), it sets $Y_{i}=\beta_{i}$, where $\beta_{i}$ is randomly chooses from $Z_{q}^{*}$.
3. Sends the system public parameters $\left\{Y,Y_{i},i\in U\right\}$ to A and keeps the secret parameter yi as secret.
  In the above scenario, A does not observe any change as $\left\{Y,Y_{i}\right\}$ and $y_{i}$ are analogous to $\left\{PK,PK_{i}\right\}$ and $\beta_{i}$ of the proposed scheme.

**Phase 1:**A adoptivily calls for a number of secret key components $K_{A}^{1},K_{A}^{2},\ldots ,K_{A}^{n}$ of attribute sets ${℧}_{1},{℧}_{2},\ldots ,{℧}_{n}$ such that all the attribute sets associated to the corresponding secret key components do not satisfy the $T^{*}$. Now, $S_{\beta }$ sends the secret key components $K_{i}$ to A as follows:

**Case 1**. if i∈℧, it sets $K_{i}$ as

(1) $$K_{i}=H\left(GID\right)\cdot \alpha^{t}\cdot r_{i}^{-1}$$

**Case 2**. if i∈(U−℧), it sets $K_{i}$ as

(2) $$K_{i}=H\left(GID\right)\cdot \alpha^{t}\cdot {\left(\beta_{i}\cdot d\right)}^{-1}$$

The distribution for both the terms in Equations (1) and (2) is uniform, thus, in A’s perspective, the key components generated by $S_{\beta }$ are the same as the basic scheme.

**Challenge:**A submits two equal length messages $M_{0}$ and $M_{1}$ to $S_{\beta }$. First $S_{\beta }$ sets $T^{*}=T_{local}^{*}⋀T_{esp}^{*}$ and then sends $T_{local}^{*}$ to the DO. It randomly selects $S,S_{1},S_{2}\in Z_{q}^{*}$ and sets $q_{R}\left(0\right)=S$ for root node R according to the proposed scheme. $S_{\beta }$ is also sent $T_{esp}^{*}$ along with $S_{1}$ to ESP (i-e sink node) to distribute it for the remaining attributes in $T^{*}\cdot S_{\beta }$ randomly selects a bit b∈{0,1} to encrypt $M_{b}$ and generates the ciphertext $CT^{*}$ as follows:

$$SK_{S_{\beta }}=S\cdot Y=\left(S_{x},S_{y}\right)$$

Hence, $S_{x}$ and $S_{y}$ represent the encryption and integrity K for message M, respectively. Afterwards, $S_{\beta }$ computes $C_{i}^{\prime }=r_{i}\cdot B$.

$S_{\beta }$ after computing $C_{S_{\beta }}=Enc\left(M_{b},S_{x}\right)$ and $INT_{M_{b}}=HMAC\left(M_{b},S_{y}\right)$ transmits below ciphertext to adversary A.

$$CT^{*}=\left(T^{*}=T_{local}^{*}\cup T_{esp}^{*},C_{S_{\beta }},INT_{M_{b}},C_{i}^{\prime }\right)$$

The challenger C flips coin μ∈{0,1}, thus the following cases arises:

- If μ=0; satisfies case 1, which is identical to our original encryption, then Z=c·d·G. Therefore, if S is set to d, there should be $SK_{S_{\beta }}^{\prime }=d\cdot Y=d\cdot c\cdot G=Z$, and $C_{i}=q_{x}\left(o\right)\cdot Y_{i}=d\cdot Y_{i}=d\cdot r_{i}\cdot G=r_{i}\cdot B$, where i∈℧.
- If μ=1; satisfies case 2, which is different from our proposed scheme, then Z=z·G. Therefore, if S is set to z, it turns out that $SK_{S_{\beta }}^{\prime }=z\cdot Y=z\cdot c\cdot G=Z$, and $C_{i}=z\cdot Y_{i}=z\cdot r_{i}\cdot G=r_{i}\cdot Z$.

**Phase 2:** Both A and $S_{\beta }$ follow the same steps as they did in Phase 1.

**Guess:**A output a guess $b^{\prime }$ of *b* to $S_{\beta }$.

- If $b^{\prime }=b$, $S_{\beta }$ output $\mu^{\prime }=0$, which indicates a valid ECDDH instance, (A,B,Z)=(c·G,d·G,c·d·G).
- If $b^{\prime }\neq b$, $b^{\prime }$ output $\mu^{\prime }=1$, which indicates a random instance, (A,B,Z)=(c·G,d·G,z·G).

Now, according to the security game, where μ=1, the adversary A cannot predict the $M_{b}$, thus we have

(3) $$Pr\left[\mu =1|b^{\prime }\neq b\right]=\frac{1}{2}$$

Since $S_{\beta }$ outputs $\mu^{\prime }=1$ when $b^{\prime }\neq b$, it gives

(4) $$Pr\left[\mu^{\prime }=\mu |\mu =1\right]=\frac{1}{2}$$

When μ=0, the adversary A can predict the correct $M_{b}$, thus we have

(5) $$Pr\left[\mu =0|b^{\prime }=b\right]=\frac{1}{2}+\epsilon$$

Since $S_{\beta }$ outputs $\mu^{\prime }=0$ when $b^{\prime }=b$, we have

(6) $$Pr\left[\mu^{\prime }=\mu |\mu =0\right]=\frac{1}{2}+\epsilon$$

According to the selective set security model of our proposed scheme, the overall advantage using Equations (8) and (10) of $S_{\beta }$ in this game is

$$\epsilon^{\prime }=\frac{1}{2}Pr\left[\mu^{\prime }=\mu |\mu =0\right]+\frac{1}{2}Pr\left[\mu^{\prime }=\mu |\mu =1\right]-\frac{1}{2}$$

or,

$$\epsilon^{\prime }=\frac{1}{2}\left(\frac{1}{2}+\epsilon \right)+\frac{1}{2}\left(\frac{1}{2}\right)-\frac{1}{2}$$

or,

$$\epsilon^{\prime }=\frac{1}{4}+\frac{\epsilon }{2}+\frac{1}{4}-\frac{1}{2}$$

or,

$$\epsilon^{\prime }=\frac{\epsilon }{2}$$

Hence, it conflicts with our assumption, which proves the security of our proposed scheme under the ECDDH assumption. □

### 5.2. Secure against Collusion Attack

One of the most anticipated attacks on any attribute-based system is a collision attack. Therefore, it is required of the designers of such a system to implicitly avoid it in their proposed scheme. Let us assume that multiple users possess some secret key components, where no individual secret key has access to the message. If they play the role of an attacker to launch a collision attack (i.e., a combination of their secret keys) by trying to decrypt a message that is encrypted under the intersects (common attributes) of their attributes sets. It is assumed that they constitute secret key components labeled to their common attribute set in the form of

$$SK_{u}=\left(K_{0}=\left\{\alpha^{1-t}\right\},K_{i}=H\left(GID\right)\cdot \alpha^{t}\cdot \beta^{-1}\right)$$

Even after collectively generating secret keys among themselves, still, they are unable to decrypt the message because of the random selection of GID for each user to satisfy the equation

$$\frac{K_{i}\cdot C_{i}}{H(GID)}\;\forall \;i\;\in ℧$$

Hence, the association of the secret key component with attributes along with a unique global identity GID and a random number $t\in Z_{p}^{*}$ for each user makes the proposed scheme resistant to collusion attack.

### 5.3. Attribute/User Revocation

Nowadays, revocation is a desirable property on the part of an ABE-based scheme. Considering the following aspects, equipping the ABE scheme with revocation is not a simple task: First, the attribute authority labeled each user secret key from a universal set of attributes instead of a unique user-specific attribute. As a result, a malicious user cannot simply be singled out on an attribute or set of attributes; second, after the revocation of a misbehaving user, the system must avoid the collusion attack even if there exists the overlapping of attributes with non-revoked users. The ABE scheme supports two types of revocation, direct revocation and indirect revocation, to address these issues. Indirect revocation incurs the liability on TAA to update and distribute the non-revoked users’ secret key with every revocation event. In direct revocation, we do not need to perform updation on the secret key of non-revoked users. All contemporary direct revocation schemes require system users to maintain an updated and long list of revoked users, which must be labeled to ciphertext. This computation and storage overhead linearly increases with the increase in revoked users in the encryption and decryption algorithms system.

Given the resource-constrained and medical-centric characteristics of our proposed scheme MAA, the indirect revocation fits aptly into our ehealth practical scenario. The computation and storage cost of our scheme is independent of the number of revoked users. The KGC of MAA explicitly maintains the list of global IDs GID and its associated attribute lists for each registered user. To revoke the system attribute from its universal set of attributes, the KGC deletes the associated system attribute’s public key. Similarly, to revoke the user-specific attribute, the KGC must delete the corresponding secret key component for that specific user. Further, KGS deletes the entire attribute set and the GID assigned to that user to revoke a user. For all of these revocation scenarios, the MAA needs to update the delegated key DK with the help of MSK and the revoked ${\mathrm{DK}}_{\beta }$ of the revoked attribute β and produces a new delegate key ${\mathrm{DK}}_{\beta^{*}}$ of the revoked attribute β. Furthermore, our proposed scheme avoids the need for maintaining a private channel between the MAA and the non-revoked user for the dissemination of the updated delegated key ${\mathrm{DK}}_{\beta^{*}}$.

## 6. Performance Analysis

In this section, we compare our proposed scheme with five related schemes in [19,20,21,22,23], in terms of its features, communication overhead, and computation overhead. Moreover, for the sake of fair comparison, we set n = 20 and m = 10 representing attributes in universal set and encryption, respectively.

### 6.1. Features Analysis

Table 2 depicts the comparison of various features of our scheme with related schemes for a WBAN from four perspectives: encryption delegation, decryption delegation, integrity check, and attribute revocation. Additionally, our proposed scheme lacks time-based access control and hierarchical access control support. In some practical scenarios, it is inevitable to provide access control for a specific time interval. For instance, a medical document may have different privacy requirements for a different period. More specifically, fewer medical experts have access to the medical record at an early time, while more experts can get access to it at a later time point. Similarly, the hierarchical access permission ensures access to the corresponding documents based on the specific role of the data users. For example, the hospital president can access all the information of the patients and doctors, while the medical experts can access his/her patient information only.

### 6.2. Communication Overhead

Communication overhead relates to the transfer of the message. In the most commonly adopted architectures of ABE, the least number of messages that should be transmitted are of the public key, private key, and ciphertext. For the sake of analysis, we take the length of these messages as a metric to determine and compare the relative communication overhead. Most contemporary ABE schemes use bilinear pairing; a map involves two groups $G_{1},G_{T}$. Because of the underlying modular exponentiation, these are termed RSA-based ABE schemes. Accordingly, we call our scheme an ABE ECC-based scheme.

As we know, ECC has much stronger hit security; we considered 160-bit, i.e., secp160r1 elliptic curve, which has up to 1024-bit RSA security strength. Based on the above-stated assumptions, the size of both public and private keys in the ABE RSA-based scheme is 1024-bit, while the size of an element in $G_{1}$ and $G_{T}$ is 1024 bits and 2048-bits. Accordingly, the size of an elliptic curve point is 320 bits, corresponding to both its coordinates. As a result, the 160 bits and 320 bits constitute the private key and public key size, respectively, in ABE ECC-based schemes. For comparison, the communication overhead is identical for each ABE RSA-based scheme. Therefore, we compute the [23] overhead for illustration purposes. The ciphertext in [23] scheme is given by $CT=(C=Me{\left(g,g\right)}^{\alpha s},C^{\prime }=g^{s},\left\{C_{i}=g^{a\lambda_{i}}g^{-r_{i}H(att_{\left(i\right)}},D_{i}=g^{r_{i}}|i\in m\right\})$, where m represents the maximum number of attributes attached to the ciphertext. According to the setup phase of this scheme, g and e(g,g) belong to the group $G_{1}$ and $G_{T}$, respectively. As a result, the size of each ciphertext component $C,C^{\prime },C_{i}$ and $D_{i}$ is 2048, 1024, (2m × 1024) and (m × 1024) bits, respectively. In this way, the length of ciphertext CT is (3m + 3) × 1024 ≈ 33,792 bits. Here, the public key is set to $PK=\{g,e{\left(g,g\right)}^{\alpha },g^{\alpha },H\}$, so its length is 4 × 1024 ≈ 4096 bits. In addition, the private key is given by $K=(g^{\alpha },l=g^{t},\left\{K_{x}=g^{H(att_{\left(x\right)}t}|x\in S\right\})$ where S represents the user set of attributes associated to the key K. Therefore, the length of the private key of scheme [23] computes to (m + 3) × 1024 ≈ 13,312 bits.

Similarly, we compute the public key, private key, and ciphertext length in our scheme. According to the encryption process of our proposed scheme, the ciphertext is $CT=(T,C_{m},INT_{m},C_{y}=q_{y}\left(0\right)\cdot PK_{y}|y\in T)$. The size of attribute set T is taken constantly for all schemes and, hence, rolled out of the total ciphertext size. Here, $C_{m}$ and $INT_{m}$ are the single coordinates on the elliptic curve, each having 160 bits in length. Similarly, $C_{y}$ consists of 320 bits, a single point on the elliptic curve. Thus, the length of the ciphertext in our proposed scheme computes to (m + 1) × 320 ≈ 3520 bits. The public key components in our scheme are $\left(PK,\left\{PK_{i}|i\in U\right\}\right)$, and consists of (n + 1) × 320 ≈ 6720 bits, as each of its components is a single point on the elliptic curve. The private key of our scheme is $K_{local}=\left(\alpha^{1-t}\right)$, $\mathrm{DK}=(\left\{\forall i\in S_{u}:K_{i}=H\left(GID\right)\cdot \alpha^{t}\cdot \beta_{i}^{-1}\right\})$. Hence, its length computes to (m + 1) × 160 ≈ 1760 bits.

We can see from Table 3 that the ciphertext and private key sizes of our proposed scheme are significantly lower than those of all other schemes. We can observe from Table 3 that only the length of the public key in our proposed scheme is higher than the scheme with a constant-size public key [19,23]. However, overall communication overhead for the private key, the public key, and ciphertext size in our scheme is significantly lower than that of [19]. Moreover, the scheme in [23] is based on KP-ABE as opposed to our CP-ABE-based scheme, which provides more control to the patient over the recipient of its sensitive medical data. Moreover, the generation of the public key is a one-time process in the lifetime of the system.

### 6.3. Computation Overhead

The computation overhead is mainly caused by the ABE scheme operations, including bilinear pairing, ECC-based scalar point multiplication, exponentiation, hashing, basic arithmetic, and logical operations. We have considered the most expensive exponentiation operations, bilinear pairing, and elliptic curve base scalar point multiplications. Comparatively, the cost of other least costly operations can be ignored [3]. For the sake of simplicity, Table 4, based on [37], is constructed, which shows the execution time (in millisecond) required by each group operation. According to work in [37], single bilinear pairing and modular exponentiation operation is about 10 and 2 times ECC-based scalar point multiplication, respectively.

To evaluate the computation overhead of the proposed scheme, we need the individual computation overhead of users and service providers on both the encryption and decryption sides. Therefore, in Table 5, we compare the computation overhead incurred on MDO and ESP in the encryption offloading and the MDU and DSP in the decryption offloading. As our scheme is free from costly pairing operations, all matrices’ execution time is comparatively less than other schemes. We can also see from Table 5 that the unwanted linearity property of ABE is shifted to comparatively resource-rich server providers (DSP and ESP). Hence, the data users are left with a significantly less and constant number of operations. Thus, based on the performance assessments, our scheme demonstrates more efficiency and the best solution for a WBAN in terms of communication, computation, and security.

### 6.4. Rank-Based Evaluation of Performance Matrices

In this research work, a fuzzy logic-based evaluation, which is constructed on the method distance from average solution (EDAS), is used for calculating the ranking of the proposed scheme with state-of-the-art algorithms in terms of computational cost operations, such as KeyGen, Enc${}_{Local}$, Enc${}_{Out}$, Dec${}_{Local}$, and Dec${}_{Out}$, on both the sides of the sender and receiver to find the top rank efficiency of these schemes. The above-stated performance matrices/operations are compared with existing state-of-the-art schemes, including the proposed scheme in this section.

In this evaluation, the authors use the EDAS approach to collect the cross-efficient values of numerous parameters of five schemes, including the proposed scheme. The aggregate of appraisal scores (λ) can be measured for ranking of given schemes to compute the positive distance from the average solution, which is represented in the equation as ($P_{I}$) and the negative distance from the average solution is represented by the symbol ($N_{I}$).

In Table 6 below, the performance matrices are deliberated as the criteria of state-of-the-art schemes.

**Step 1:** Calculate the solution of the average value (ψ ) of all matrices in Equation (7);

(7) $$\left(\psi_{\beta }\right)\;={\left[\psi_{\beta }\right]}_{1\times \delta }$$

where,

(8) $$\left(\psi \right)=\frac{\sum_{i=1}^{x}X_{\alpha \beta }}{x}$$

The above steps define the performance matrices as benchmarks of various schemes. The calculation of aggregate in Equations (7) and (8) can be gained as the average value (ψ) for each calculated benchmark value against each given value in Table 7.

**Step 2:** In this step of the EDAS method, the positive distance from the average is denoted as $\left(P_{I}\right)$, and is calculated as shown in Equations (9)–(11) as given below:

(9) $$P_{I}={\left[{\left(P_{I}\right)}_{\alpha \beta }\;\right]}_{\delta \times \delta }$$

If the βth criterion is more beneficial, then

(10) $${{(P}_{I})}_{\alpha \beta }=\frac{Maximum(0,\;\;\;\left(A_{V_{\beta }}-X_{\alpha \beta }\right))}{A_{V_{\beta }}}$$

and if non-beneficial, then the given equation will be changed as follows below:

(11) $${{(P}_{I})}_{\alpha \beta }=\frac{Maximum(0,\;\;\;\left(X_{\alpha \beta }-A_{V_{\beta }}\right))}{A_{V_{\beta }}}$$

The results replicate in Table 8 following as:

**Step 3:** In this step of the EDAS, the negative distance from the average is denoted as $\;{(N}_{I}$), and is calculated using Equations (12), (13) and (15) as follows:

(12) $$\;\;\;{(N}_{I})={\left[{{(N}_{I})}_{\alpha \beta }\right]}_{\delta \times \delta }$$

If the β${}_{th}$ criterion is more beneficial, then

(13) $${{(N}_{I})}_{\alpha \beta }=\frac{Maximum(0,\;\;\;\left(A_{V_{\beta }}-X_{\alpha \beta }\right))}{A_{V_{\beta }}}$$

and if non-beneficial, then the given equation will be changed as follows below:

(14) $${{(N}_{I})}_{\alpha \beta }=\frac{Maximum(0,\;\;\;\left(X_{\alpha \beta }-A_{V_{\beta }}\right))}{A_{V_{\beta }}}$$

In the above equations, ${{(P}_{I})}_{\alpha \beta }$ and ${\;{(N}_{I})}_{\alpha \beta }$ stand for the positive distance and negative distance of β${}_{th}$ appraised algorithms from the average value concerning α${}_{th}$ rating performance parameters, respectively.

The results reproduced are shown in Table 8 as:

**Step 4:** In this step, the the weighted sum of ${(P}_{I})$ for the rated algorithms in Table 9 is shown below:

(15) $${{(SP}_{I})}_{\alpha }=\sum_{\beta =1}^{x}y_{\beta }{{(P}_{I})}_{\alpha \beta }$$

**Step 5:** In this step, the weighted sum of ${{(N}_{I})}_{\alpha \beta }$ for the rated algorithms in Table 10 is shown below in Equation (16):

(16) $${{(SN}_{I})}_{\alpha }=\sum_{\beta =1}^{x}y_{\beta }{{(N}_{I})}_{\alpha \beta }$$

The results obtained are reflected in Table 10 as shown:

**Step 6:** In this step, the normalized scores of ${{(SP}_{I})}_{\alpha }$ and ${{(SN}_{I})}_{\alpha }$ for the rated algorithms are calculated as presented in Equations (17) and (18):

(17) $$N{{(SP}_{I})}_{\alpha }=\frac{{{(SP}_{I})}_{\alpha }}{{maximum}_{\alpha }\left({{(SP}_{I})}_{\alpha }\right)}$$

(18) $$N{{(SN}_{I})}_{\alpha }=1-\frac{{{(SN}_{I})}_{\alpha }\;}{{maximum}_{\alpha }\left({{(SN}_{I})}_{\alpha }\right)}$$

**Step 7:** In this step, the scores of $\;N{{(SP}_{I})}_{\alpha }$ and $N{{(SN}_{I})}_{\alpha }$ to receive an appraisal score (*AS*) is calculated, which is equal to (λ) for the rated algorithms given in Equation (19).

(19) $$\lambda_{\alpha }=\frac{1}{2}\left(N{{(SP}_{I})}_{\alpha }-N{{(SP}_{I})}_{\alpha }\right)$$

where $0\leq \lambda_{\alpha }\leq 1$.

The (λ) is determined by the aggregate score of $NSP_{m}$ and $NSN_{m}$.

**Step 8:** In this step, measurement of the appraisal scores (λ) in terms of decreasing order and then concluding of the ranking of rated algorithms is performed. The paramount ranking algorithms have the higher (λ). Thus, in Table 11 below, the proposed algorithm has the highest (λ).

The final results of the overall ranking are represented in Table 11:

The ranking shows that the proposed algorithm is the best out of five total state-of-the-art algorithms in the stated research domain.

## 7. Conclusions and Future work

In summary, we present a secure and efficient ABE architecture with outsourcing intense encryption and delegation operations. Further, leverage on the lightweight features of ECC and the primitive syntax of CP-ABE, our scheme reduces the computation cost of both encryption and decryption on the user side into a constant. Our solution enables the resource-scarce and lightweight WBAN sensors to securely upload and retrieve sensitive medical data in public clouds with a minimum constant cost. The inherent features of attribute/user revocation and verifiability of outsourcing data further strengthen the security of our scheme. The proposed scheme is found to be secured under the ECDDH assumption using the selective-set security model. The performance assessment of our scheme shows a significant overall efficiency in terms of storage, computation, and communication. Further, for better clarification and evaluation, the final outputs of the EDAS ranking method show that the proposed approach is on the top rank that noticeably reported the proposed scheme’s outperformance than the other reference schemes. We will investigate the incorporation of time-based access control and hierarchical access control in our research work as future work.

## Figures and Tables

**Table 1:** Notations.

Notations	Description
λ	Security parameter
*P*	Represents the number of elements in the finite field $Z_{p}$
*q*	Represents the order of G in E
$E_{q}\left(a,b\right)$	An elliptic curve defined over the finite field $Z_{q}$
*G*	A base point in $E_{q}\left(a,b\right)$
$G_{E}$	A cyclic subgroup of E of order *q*
$Z_{q}$	A finite field, whose integer elements are {0,1,…,q−1}
$Z_{q}^{*}$	$Z_{q}^{*}=Z_{q}\backslash \left\{0\right\}$
K∗P	Scalar point multiplication, $P\in E_{q}$
*O*	A point at infinity of an elliptic curve group
PARAMS	The system public key parameter
SMK	Master secret key of the system
$HMAC(S_{y},M)$	Hash function to output message integrity check of *M* using integrity key $S_{y}$
$Enc(S_{x},M)$	Encryption of message *M* with symmetric key $S_{x}$
$Dec(S_{x},C_{M})$	Decryption of ciphertext $C_{M}$ with symmetric key $S_{x}$
$T_{BP}$	Time cost of bilinear pairing operation
$T_{EXP}$	Time cost of exponentiation
$T_{PM}$	Time cost of elliptic curve scalar point multiplication

**Table 2:** Features comparison.

Scheme	[19]	[20]	[21]	[22]	[23]	Proposed
Encrypt Delegation	✓	×	✓	✓	✓	✓
Decrypt Delegation	×	✓	✓	✓	✓	✓
Integrity Check	×	✓	×	×	✓	✓
Attribute Revocation	×	×	×	×	×	✓

**Table 3:** Parameters size (bits).

Scheme	Private Key Size	Public Key Size	Ciphertext Size
[19]	(2m+2)×1024≈22,522	(3×1024)≈3072	(2m+2)×1024≈22,528
[20]	(5m+3)×1024≈54,272	(n+3)×1024≈23,552	(2m+3)×1024≈23,552
[21]	(m+4)×1024≈14,336	(n+4)×≈24,576	(2m+3)×1024≈23,552
[22]	(3m+1)×1024≈31,744	(n+3)×1024≈23,552	(2m+1)×1024≈21,504
[23]	(m+3)×1024≈13,312	(4×1024)≈4096	(3m+3)×1024≈33,792
Proposed	(m+1)×160≈1760	(n+1)×320≈6720	(m+1)×320≈3520

**Table 4:** Execution time for cryptographic operations.

Operations	$T_{\mathrm{BP}}$	$T_{\mathrm{EXP}}$	$T_{\mathrm{PM}}$
Time (ms)	20.04	5.31	2.21

**Table 5:** Computational overhead (ms).

Scheme	KeyGen	Enc*_Local_*	Enc*_Out_*	Dec*_Local_*	Dec*_Out_*
[19]	$\left(1+3m\right)T_{EX}\approx 164.61$	$5T_{EX}\approx 26.55$	$2mT_{EX}\approx 106.2$	$2m(T_{bp}+T_{EX})\approx 507$	-
[20]	$\left(7m+5\right)T_{EX}\approx 398.25$	$\left(4+2m\right)T_{EX}\approx 127.44$	-	$T_{EX}\approx 5.31$	$2m\left(T_{Bp}+T_{EX}\right)+2T_{Bp}\approx 547.08$
[21]	$\left(3m+7\right)T_{EX}\approx 196.47$	$\left(2m+3\right)T_{EX}\approx 122.13$	$\left(2m+1\right)T_{EX}\approx 111.51$	$(T_{EX})\approx 5.31$	$\left(2m+1\right)T_{Bp}+\left(3m+1\right)T_{EX}\approx 584.61$
[22]	$\left(1+4m\right)T_{EX}\approx 217.71$	$(5T_{EX})\approx 26.55$	$\left(2m-2\right)T_{EX}\approx 95.98$	$(T_{BP}+2T_{EX})\approx 30.66$	$2m(T_{BP}+T_{EX})\approx 506.8$
[23]	$\left(m+4\right)T_{EX}\approx 127.44$	$4T_{EX}\approx 21.24$	$3mT_{EX}\approx 159.3$	$T_{BP}\approx 20.04$	$2mT_{EX}mT_{BP}\approx 306.4$
Proposed	$\left(m+1\right)T_{EX}\approx 111.51$	$2T_{PM}\approx 4.42$	$\left(m-2\right)T_{PM}\approx 17.68$	$T_{PM}\approx 2.21$	$2m.T_{PM}\approx 44.2$

**Table 6:** Analysis results of average.

Scheme	Performance Metrics				
	KeyGen	Enc*_Local_*	Enc*_Out_*	Dec*_Local_*	Dec*_Out_*
[19]	0.1877	0.5148	0	0	1
[20]	0	0	1	0.9441	0
[21]	0	0	0.3	0.7350	0
[22]	0	0.5148	0	0.6775	0
[23]	0.3711	0.6118	0	0.7892	0.0757
Proposed	0.4497	0.9192	0.7838	0.9767	0.8666

**Table 7:** Cross-efficient values.

Scheme	Performance Metrics				
	KeyGen	Enc*_Local_*	Enc*_Out_*	Dec*_Local_*	Dec*_Out_*
[19]	164.61	26.55	106.2	507	0
[20]	398.25	127.44	0	5.31	547.08
[21]	196.47	122.13	111.51	5.31	584.61
[22]	217.71	26.55	95.98	30.66	506.8
[23]	127.44	21.24	159.3	20.04	306.4
Proposed	111.51	4.42	17.68	2.21	44.2
$\psi_{\beta }$	202.665	54.7216	81.7783	95.0883	331.515

**Table 8:** Analysis results of average ${(N}_{I}$).

Scheme	Performance Metrics				
	KeyGen	Enc*_Local_*	Enc*_Out_*	Dec*_Local_*	Dec*_Out_*
[19]	0	0	0.2986	4.3318	0
[20]	0.9650	1.3288	0	0	0.6502
[21]	0	1.2318	0.3635	0	0.7634
[22]	0.9523	5.0067	4.4287	12.8733	10.4660
[23]	0	0	0.9479	0	0
Proposed	0	0	0	0	0

**Table 9:** Analysis results of the aggregate ${(P}_{I})$.

Criteria (W)	0.4176	0.2850	0.1453	0.0844	0.0676	${{(\mathrm{SP}}_{I})}_{\alpha }$
Scheme	Performance Metrics					
	KeyGen	Enc*_Local_*	Enc*_Out_*	Dec*_Local_*	Dec*_Out_*	
[19]	0.0784	0.1467	0	0	0.0676	0.2927
[20]	0	0	0.1453	0.0796	0	0.2250
[21]	0.0127	0	0	0.0796	0	0.0924
[22]	0	0	0	0	0	0
[23]	0.1550	0.1743	0	0.0666	0.0051	0.4011
Proposed	0.1878	0.2619	0.1139	0.0824	0.0586	0.7047

**Table 10:** Analysis results of the aggregate ${(N}_{I})$.

Criteria (W)	0.4176	0.2850	0.1453	0.0844	0.0676	${{(\mathrm{SP}}_{I})}_{\alpha }$
Scheme	Performance Metrics					
	KeyGen	Enc*_Local_*	Enc*_Out_*	Dec*_Local_*	Dec*_Out_*	
[19]	0	0	0.04340	0.3656	0	0.4090
[20]	0.4030	0.3787	0	0	0.0439	0.8257
[21]	0	0.3510	0.0528	0	0.0516	0.4555
[22]	0	0	0	0	0	0
[23]	0	0	0.1377	0	0	0.1377
Proposed	0	0	0	0	0	0

**Table 11:** Analysis results of five state-of-the-art schemes.

Scheme	${{(\mathrm{SP}}_{I})}_{\alpha }$	${{(\mathrm{SN}}_{I})}_{\alpha }$	$N{{(\mathrm{SP}}_{I})}_{\alpha }$	$N{{(\mathrm{SN}}_{I})}_{\alpha }$	$\lambda_{\alpha }$	Ranking
[19]	0.2927	0.4090	0.4154	0.5046	0.4600	4
[20]	0.2250	0.8257	0.3192	0	0.1596	6
[21]	0.0924	0.4555	0.1311	0.4483	0.2897	5
[22]	0	0	0	1	0.5	3
[23]	0.4011	0.1377	0.5691	0.8331	0.7011	2
Proposed	0.7047	0	1	1	1	1
